# Singing out of tune: sexual and developmental differences in the occurrence of nonlinear phenomena in primate songs

**DOI:** 10.1098/rstb.2024.0021

**Published:** 2025-04-03

**Authors:** Chiara De Gregorio, Daria Valente, Walter Cristiano, Filippo Carugati, Michela Prealta, Valeria Ferrario, Teresa Raimondi, Valeria Torti, Jonah Ratsimbazafy, Cristina Giacoma, Marco Gamba

**Affiliations:** ^1^ Department of Life Sciences and Systems Biology, University of Torino, Torino 10123, Italy; ^2^ Department of Psychology, University of Warwick, Coventry CV4 7A, UK; ^3^ Parco Natura Viva Garda Zoological Park (PNV), Bussolengo 37012, Italy; ^4^ Environment and Health Department, Italian National Institute of Health, Roma 00161, Italy; ^5^ Chester Zoo, Caughall Road, Chester CH2 1LE, UK; ^6^ Department of Human Neurosciences, Sapienza University of Rome, Roma 00185, Italy; ^7^ Groupe d’Etude et de Recherche sur les Primates de Madagascar, Antananarivo 779, Madagascar

**Keywords:** ontogeny, singing primates, vocalization, vocal fatigue, chaos, phonation

## Abstract

Animal vocalizations contain a varying degree of nonlinear phenomena (NLP) caused by irregular or chaotic vocal organ dynamics. Several hypotheses have been proposed to explain NLP presence, from unintentional by-products of poor vocal technique to having a functional communicative role. We aimed to disentangle the role of sex, age and physiological constraints in the occurrence of NLP in the songs of the lemur *Indri indri*, which are complex harmonic vocal displays organized in phrases. Age and sex affected the presence and type of NLP in songs. In particular, the proportion of the phenomena considered decreased with age, except for subharmonics. Subharmonics potentially mediate the perception of lower pitch, making signallers appear larger. Subharmonics and frequency jumps occurred in lower-pitched notes than regular units, while chaos and sidebands occurred in higher-pitched units. This suggests that different types of NLP can be associated with different vocal constraints. Finally, indris might present short-term vocal fatigue, with units occurring in the last position of a phrase having the highest probability of containing NLP. The presence of NLP in indris might result from proximate causes, such as physiological constraints, and ultimate causes, such as evolutionary pressures, which shaped the communicative role of NLP.

This article is part of the theme issue ‘Nonlinear phenomena in vertebrate vocalizations: mechanisms and communicative functions’.

## 1. Introduction

Nonlinear phenomena (NLP) in animal vocalizations arise from irregularities in the oscillation of the vocal folds. The production of animal vocalization typically involves oscillating vocal folds that vibrate thanks to the subglottal pressure and the Bernoulli effect [1]. Vocal nonlinearities or NLP (hereafter ‘NLP’) occur when the normal vibrational pattern of the paired laryngeal vocal folds is perturbed, caused by the asynchronization of their oscillation [2]. Perceptually, we can associate NLP with vocalizations showing a variable degree of harshness. Depending on the perturbation of the vocal folds’ cycling, this process can result in different types of NLP, such as frequency jumps, subharmonics, sidebands, and deterministic chaos [2–4]. *Frequency jumps* are abrupt breaks in the fundamental frequency, while the vocal folds create *subharmonics* when they move with different frequencies. In this case, the vocal folds are partially synchronized. In contrast, sidebands for each harmonic appear in the spectrogram when one fold vibrates with a much lower frequency than the other or additional oscillators, such as lips or flaps above the larynx, vibrate [5,6].

Increasing evidence from a wide range of animal species shows that changes in the harmonic structure of sounds represent a source of acoustic variation that can encode information about the emitter, capture the listener’s attention, and avoid habituation to vocalizations. For example, studies on NLP in rhesus macaques (*Macaca mulatta*) and dholes (*Cuon alpinus*) suggested that they can inform about the identity of the vocalizing individual [3,7]. In whales (*Megaptera novaeangliae*), NLP have been linked to fitness and body size signalling [8]. Moreover, nonlinear sound production may signal increased arousal in red wolves (*Canis rufus*) [9]. On the other hand, it is not clear whether the sex of the emitter plays a role in the occurrence and structure of NLP. Only some studies have investigated this aspect, showing mixed evidence. For example, no sexual differences emerged in the occurrence of NLP in dholes [10], while female golden hamsters (*Mesocricetus auratus*) were more likely to produce a higher number of chaotic calls and calls containing frequency jumps than males [11]. Differently, male Chilean tree iguanas (*Liolaemus chiliensis*) produce fewer NLP than females [12]. Experimental evidence also showed that vocal nonlinearities in marmot calls (*Marmota flaviventris*) and singing mice pups (*Scotinomys* spp.) play a role in capturing the listener’s attention and avoiding receiver habituation [13,14]. Townsend & Manser also highlighted NLP’s attention-grabbing function in alarm calls of meerkats (*Suricata suricatta*) [15].

Furthermore, NLP play an important part in human vocal communication. Vocal nonlinearities are present in the human voice from birth [16], in verbal communication, nonverbal communication, and singing [17,18]. NLP can be related to pain perception [19] and can have positive and negative valence when expressing emotion, depending on the communication context [20]. NLP are biologically relevant in that they can convey information even across species. For example, humans can detect vocal nonlinearities in puppy whines [21].

Age is one of the factors that has been linked to the presence of NLP in animal vocal communication. In humans, the voice of adolescent males is recognizable by its frequency jumps [18,22], and nonlinear dynamics of the vocal folds may increase with ageing [23]. Non-human mammals differ in types and quantities of NLP based on their age. For example, Volodin *et al.* [24] investigated the presence of NLP during the ontogeny of yellow steppe lemmings (*Eolagurus luteus*). These authors found that NLP were most frequent in younger individuals, with some types of NLP being present only in specific age classes. Similarly, the vocal repertoire of neotropical singing mice pups (*Scotinomys* spp.) presents notes with nonlinear elements that are rare or absent in adults [14]. NLP are also common in vocalizing Asian elephant calves (*Loxodonta africana*) [25]. In dogs (*Canis lupus familiaris*), a higher occurrence of NLP in older individuals might be a potential ageing indicator [26], while in bottlenose dolphins (*Tursiops truncatus*), age is negatively correlated with nonlinearities [27]. In the begging calls of African penguins (*Spheniscus demersus*), the number of NLP increases with age in the first 2 months of life [28]; moreover, both the number and the duration of NLP are higher in individuals with respiratory disease [28].

This wide range of examples highlights the widespread occurrence of NLP in animal vocalizations across different species and contexts. Studying these phenomena offers valuable insights into the intricate dynamics of sound production in animals and can provide an understanding of the evolution of acoustic communication systems. Various hypotheses have been formulated to explain the occurrence of NLP, and animals producing harmonic vocal emissions are ideal models for studying NLP because these phenomena appear in various forms. Singing primates, in particular, are excellent candidates for such investigation for two main reasons. The first is the production of elaborated harmonic vocalizations that occur in both male and female individuals [29], making them suitable for research on sexual dimorphism in vocal nonlinearities in the animal kingdom. The second reason is their phylogenetic relationship with humans: on the one hand, the human larynx has evolved to allow the production of sounds with less aural chaos [30]; on the other hand, some primates phylogenetically distant from modern humans also communicate by emitting elaborate series of harmonic sounds. Investigation into the reason behind singing primates' vocal nonlinearities may offer insights into the presence of NLP in human communication.

The lemur *Indri indri* is the only strepsirrhine primate (i.e. lemurs, bush babies, pottos and lorises) showing singing behaviour. It sings in loud duets and choruses performed by members of a family group in the form of a series of frequency-modulated units (also termed notes) organized in phrases [31]. Recent investigation on this topic found that similarly to human speech—which occasionally can contain vocal nonlinearities [32]—most notes emitted were purely harmonic in structure, with only around 28% of notes containing NLP. Such phenomena are more likely to occur in the later stages of an indri song, possibly owing to the animals’ fatigue [33]. This suggests that indris may be subject to physiological constraints during the singing process, which impair the production of harmonic sounds. Indeed, as in chimpanzees, humans and rock hyraxes, the effort required to engage in intense and prolonged vocal activity could easily lead to vocal fold instability [34–37]. However, it remains an open question which other factors concur with the appearance of vocal nonlinearities in the indris' songs and how they are related to the emergence of a specific type of NLP rather than another.

Our work aims to investigate the NLP repertoire of indris’ songs, particularly the effect of age and sex on the presence and characteristics of different types of vocal nonlinearities. In particular, we investigated (i) the effect of sex and age on the overall number of NLP in a song and the proportion of different NLP types; (ii) whether the occurrence and the types of NLP would correlate with spectro-temporal notes’ features; and (iii) the presence of short-term vocal fatigue, by exploring whether the position of a note within a phrase influences the presence and duration of NLP. Our work will help to disentangle the role of sex, age, and physiological constraints in the occurrence of NLP.

## 2. Methods

### (a) Subjects and recordings

**Figure 1.**
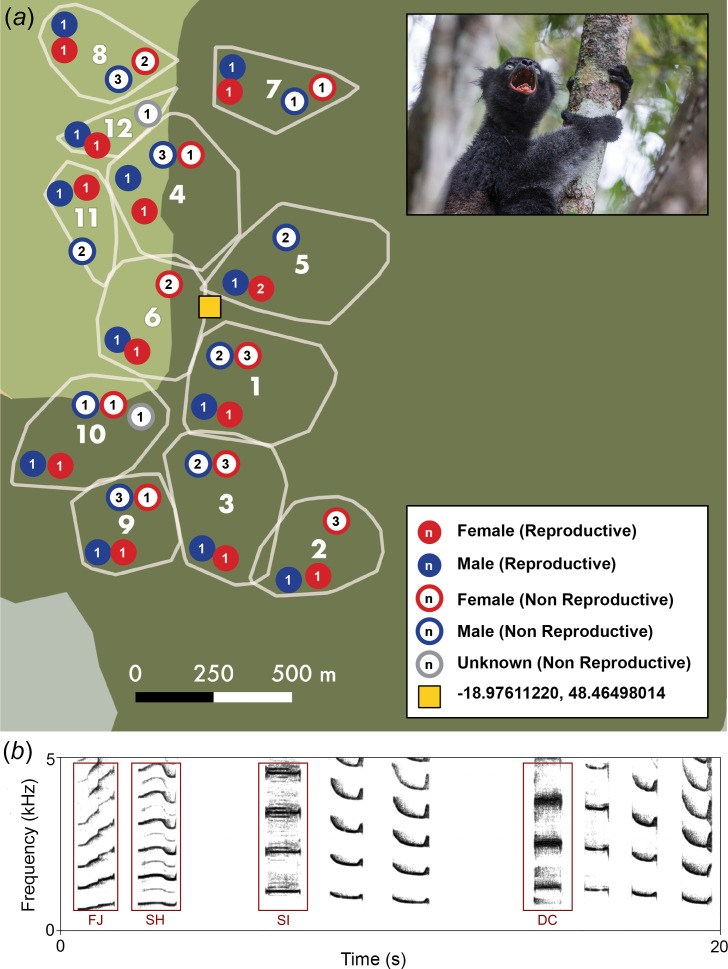
Recording site, group distribution and composition, and spectrograms of the nonlinear phenomena (NLP) in the indris. (*a*) Map showing the 12 groups of indris recorded for this study and their distribution in the Maromizaha Forest New Protected Area. Numbers refer to the group ID and are placed within a schematized minimum convex polygon estimation showing the position of group territory in the forest. Small numbers within circles refer to the number of individuals per class (males/females/unknown, reproductive/non-reproductive). A juvenile male from Group 11 (courtesy of Filippo Carugati) is shown in the inset. Yellow square represent the location of the Maromizaha Research Center. (*b*) Types of NLP present in the indri songs. Left to right: a phrase composed of two units (DP2) containing a frequency jump (FJ) in the first unit and subharmonics (SH) in the second; a phrase containing three units (DP3) showing sidebands (SI) in the first unit; a DP4 containing chaos (DC) in the first unit. The audio file containing the pictured sounds is available in the supplementary material. We generated the spectrograms using Praat [47], with the following Paint settings: time range: 0−20 s, frequency range 0−5000 Hz, maximum dB Hz^−1^ = 150, dynamic range = 50 dB, pre-emphasis = 1 dB oct^−1^, dynamic compression = 0.7, autoscaling = ‘yes’, garnish = ‘yes’.

Indris are strepsirrhine primates living in the eastern rainforest of Madagascar. They are monogamous [38], live in family groups [39], and although possessing a rich vocal repertoire [40,41] they are the only singing lemur, and use songs to defend their territory through intense singing behaviour by two or more individuals simultaneously [31,42]. Songs also exchange information about group location and composition [43,44] and are routinely given each morning [45]. Indris’ songs last, on average, 112.83 ± s.d. 53.46 s, and contain 24.69 ± s.d. 9.03 notes [46]. We recorded spontaneous songs of 62 wild indris belonging to 12 groups habituated to human presence living in the Maromizaha New Protected Area, Madagascar (18°56′49′′ S, 48°27′53′′ E; figure 1*a*) between 2014 and 2023. Songs were recorded at a 2−10 m distance using different recorders (Sound Devices 702, Olympus S100 and LS05, and Tascam DR-100, DR-40, and DR-05; sampling rate of 44.1 kHz and 16-bit resolution) connected to shotgun microphones (Sennheiser ME 66, ME 67 and AKG CK 98) oriented towards the vocalizing animals.

We obtained 831 individual contributions (the individual vocal output in a duet/chorus) from 290 duets and choruses. Of the 62 indris, 24 were reproductive adults, and 38 were their offspring (16 females, 20 males, 2 individuals for which the sex determination was impossible). For this study, we considered only the offspring for which we knew or could estimate the date of birth. We provide an accurate birthdate for the offspring we observed from the day of birth. Differently, we provide an estimated birthdate for the newborns we found during the regular sampling of the family group, setting the estimated birth date to the 15th of the actual birth month, with an accuracy of ±15  days [48]. The age of the vocalizing animal was calculated as the age (in days) at the date of song recording. The youngest individual in our dataset was 297 days (*ca* 9 months), and the oldest was 2825 days (*ca* 7.85 years). We provide details on the group composition, identity, sex and age of the individuals in electronic supplementary material, table S1.

### (b) Acoustic analyses

We used Praat software [47] to visually inspect each song’s spectrogram. We identified each indri’s individual contribution to a duet or a chorus using annotations in Praat TextGrids. We also labelled the songs’ units according to their type and position (e.g. whether units were part of a phrase [48]). If units were not part of a phrase, their code was LN for long notes and SN for single notes. If units were part of a phrase, their label considered the number of units in the phrase, the position of the unit within the phrase, and the position inside the individual contribution. We noted the presence/absence of nonlinear elements (subharmonics, frequency jumps, sidebands, deterministic chaos [3] and their duration for each unit (figure 1B)). We then isolated each unit’s fundamental frequency and saved it into a single audio file (.wav format).

We used a custom Python (version 3.9.6) [49] script to extract the duration of the unit and that of the NLP. We then used a custom script in Praat to extract a pitch point for each 0.015 ms from each unit. Finally, we used R (version 4.3.3) [50] to compute the maximum and minimum of the fundamental frequency (max *f*_o_ and min *f*_o_, respectively).

### (c) Statistical analyses

We ran seven generalized linear mixed models (GLMM; *glmmTMB* package [51] and four linear mixed models (LMM, *lme4* package [52]) with a full-vs-null approach [53]. For each model, we checked overdispersion via the *DHARMa* package (testOverdispersion function) [54]. We verified the normality and homogeneity of residuals by inspecting the *qqplot* and the residuals’ distribution (through a function provided by R. Mundry, Georg-August-Universität Göttingen). When used as a fixed factor, the variable age was *z*-transformed. We used the animal identity as a random factor in all models.

#### (i) The effect of sex and age on the occurrence of nonlinear phenomena and their types

In the first GLMM, we used the normalized count of NLP within an individual contribution as the response variable and the kind of NLP and the emitter’s sex as fixed factors. We then ran two *post hoc* tests (*emmeans* package [55]), one for performing all pairwise comparisons for the type of NLP and one for the interaction between NLP type and sex.

In the second GLMM, we used the normalized count of NLP within an individual contribution as a response variable, and the age and sex of the emitting animal as fixed factors. We corrected the model for zero inflation, using a beta distribution as suggested by the package *fitdistrplus* [56] as a suitable theoretical distribution.

We then ran three GLMMs to investigate whether the proportion of different types of NLP varied with age. In all three models, we used the normalized count of different NLP types as a response variable, obtained by dividing the number of a specific NLP type in an individual contribution by the total number of NLP present in the same contribution. We used the sex and age of the individual in interaction with the NLP type as a fixed factor. Since we were also interested in understanding if the relationship between the individual’s age and the different NLP types emitted also varied with the sex of the animals, we fitted the other two models on two subsets of data, one containing only females and the other containing only males. The rest of the syntax was the same as the first model. For all three models, we then used the function emtrends (*emmeans* package [55]) to explore the interaction between age and the type of NLP.

#### (ii) Occurrence and types of nonlinear phenomena based on spectro-temporal features

We used a sixth GLMM to understand whether the presence of NLP was related to the unit’s spectro-temporal characteristics, predicting that NLP would be associated with longer and high-pitched notes. The response variable had a binomial distribution (presence/absence; family = binomial). We used the minimum value of fundamental frequency (min *f*_o_), the maximum value of fundamental frequency (max *f*_o_), and the unit duration as fixed factors.

We also ran three LMMs to investigate the effect of the type of NLP and the sex of the emitter on, respectively, the maximum and minimum value of the fundamental frequency of units and their duration. We log-transformed min *f*_o_, max *f*_o_ and note duration and used them as response variables. For all three models, we used the interaction between the type of NLP and the sex of the emitter as a fixed factor. We then used a *post hoc* test (*emmeans* package [55]) to perform all pairwise comparisons of the type of NLP and the interaction between NLP type and sex.

#### (iii) Short-term vocal fatigue: notes position and presence and duration of nonlinear phenomena

We ran an additional GLMM to investigate the presence of NLP based on the relative position of a unit within a song (e.g. first note in a phrase, intermediate position within a phrase, last position of a phrase, isolated note not being part of a phrase) and the sex of the emitter. Also, in this case, the response variable had a binomial distribution (presence/absence; family = binomial), and we used the interaction between note position and the sex of the emitter as a fixed factor.

Lastly, the last LMM tested whether the duration of NLP (normalized for the duration of the note) was linked to the relative position of a unit within a song (e.g. first note in a phrase, intermediate position within a phrase, last position of a phrase, isolated note not being part of a phrase) and the sex of the emitter. We entered the normalized duration of NLP as the response variable and the note position and sex of the emitter as interacting fixed factors. In this model, we used as nested random factors the individual identity and the type of phrase organization (e.g. the information on whether the note containing the NLP was an isolated note or belonged to a descending phrase composed of two, three, four, five and six notes: DP2, DP3, DP4, DP5, and DP6, respectively). We used two *post hoc* tests (emtrends function, package *emmeans* [55]) to compare the fixed factors pairwise.

## 3. Results

### (a) Nonlinear phenomena repertoire

Of the 831 individual contributions, 628 contained at least one nonlinear phenomenon. We isolated 16 413 vocal units from the individual contributions, mostly without nonlinearities (13 187). Out of the remaining 3226 units (23.5%), we mainly found subharmonics (*n* = 2898; 1969 in male units, 929 in female units), followed by sidebands (*n* = 208; 119 in male units, 89 in female units), frequency jumps (*n* = 71; 67 in male units, 4 in female units) and deterministic chaos (*n* = 49; 39 in male units, 10 in female units). A single individual contribution contained up to 26 nonlinear elements. On average, subadult females emitted 2.21 elements with nonlinearities per contribution, adult females emitted 3.81 elements with nonlinearities, subadult males emitted 4.27 elements with nonlinearities, and adult males emitted 5.36 elements with nonlinearities. We found the highest number of NLP in the phrases composed of three units (*n* = 1338), followed by phrases consisting of two units (*n* = 857), phrases composed of four units (*n* = 589), single units (*n* = 314) and phrases consisting of five units (*n* = 79). Long notes and phrases composed of six units contained a nonlinear phenomenon in 46 cases and two cases, respectively.

### (b) The effect of sex and age on the occurrence of nonlinear phenomena and their types

#### (i) Overall number of nonlinear phenomena, sex and age differences

**Figure 2.**
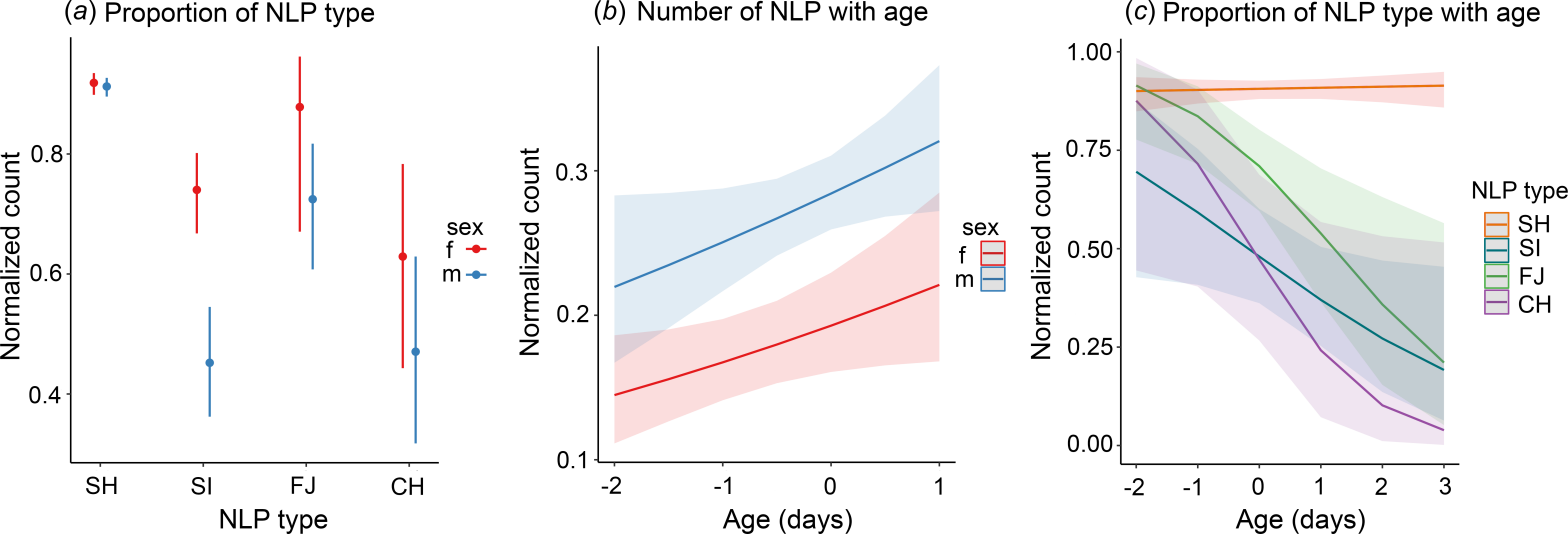
Effect of age on type, number and proportion of NLP. (*a*) Proportion of different nonlinear phenomena (NLP) types in the two sexes. (*b*) Variation of the number of NLP notes with age. (*c)* Variation in the proportion of different NLP types with age. SH, subharmonics, SI, sidebands, FJ, frequency jumps, CH, chaos. We used the R packages *ggplot2* [57] and *ggeffects* [58] for visualizing data.

We found that both age and sex had a significant effect on the overall number of NLP in the indris’ songs (full vs null model: *χ*^2^ = 48.140, d.f. = 2, *p* < 0.001). In particular, indris emitted a higher number of NLP with growth (age; estimate = 0.132, *z* = 2.407, *p* = 0.016; figure 2*b*), and male songs had more NLP than females’ (males vs females; estimate = 0.512, *z* = 4.012, *p* < 0.001; electronic supplementary material, table S2).

#### (ii) Nonlinear phenomena repertoire and sex differences

Depending on the phenomenon, we found that NLP proportion significantly differed between the sexes (full vs null model: *χ*^2^ = −5898.260, df = 6, *p* < 0.001). Subharmonics are the most frequent type of NLP, followed by frequency jumps and chaos/sidebands. We found no difference between the proportion of notes containing chaos and those containing sidebands (*p* = 0.938, electronic supplementary material, table S2; figure 2*a*). Moreover, females produced more notes containing sidebands compared with males (*p* < 0.001, figure 2*a*).

#### (iii) Type of nonlinear phenomena, variation per type with age and sex differences

The NLP proportion showed significant variation during growth, depending on the phenomenon (full vs null model: *χ*^2^ = −907.3711, df = 8, *p* < 0.001). The proportion of subharmonics produced remained stable during ontogeny (*p* = 0.718), while indris' notes had lower deterministic chaos, frequency jumps, and sidebands with growth (*p* = 0.048, *p* = 0.004 and *p* = 0.042, respectively; electronic supplementary material, table S2; figure 2*c*).

When investigating differences between the sexes, the full model, including only females, did not differ from the null one (*χ*^2^ = −569.8194, df = 5, *p* = 0.070). On the other hand, the full model including only males did (*χ*^2^ = −1272.830, df = 7, *p* < 0.001) and showed that males decreased the proportion of frequency jumps with age (*p* < 0.002, electronic supplementary material, table S3), while the proportion of sidebands, subharmonics and chaos did not vary (electronic supplementary material, table S3).

### (c) Occurrence and types of nonlinear phenomena based on spectro-temporal notes’ features

#### (i) Occurrence of nonlinear phenomena based on note duration, maximum and minimum fundamental frequency

**Figure 3.**
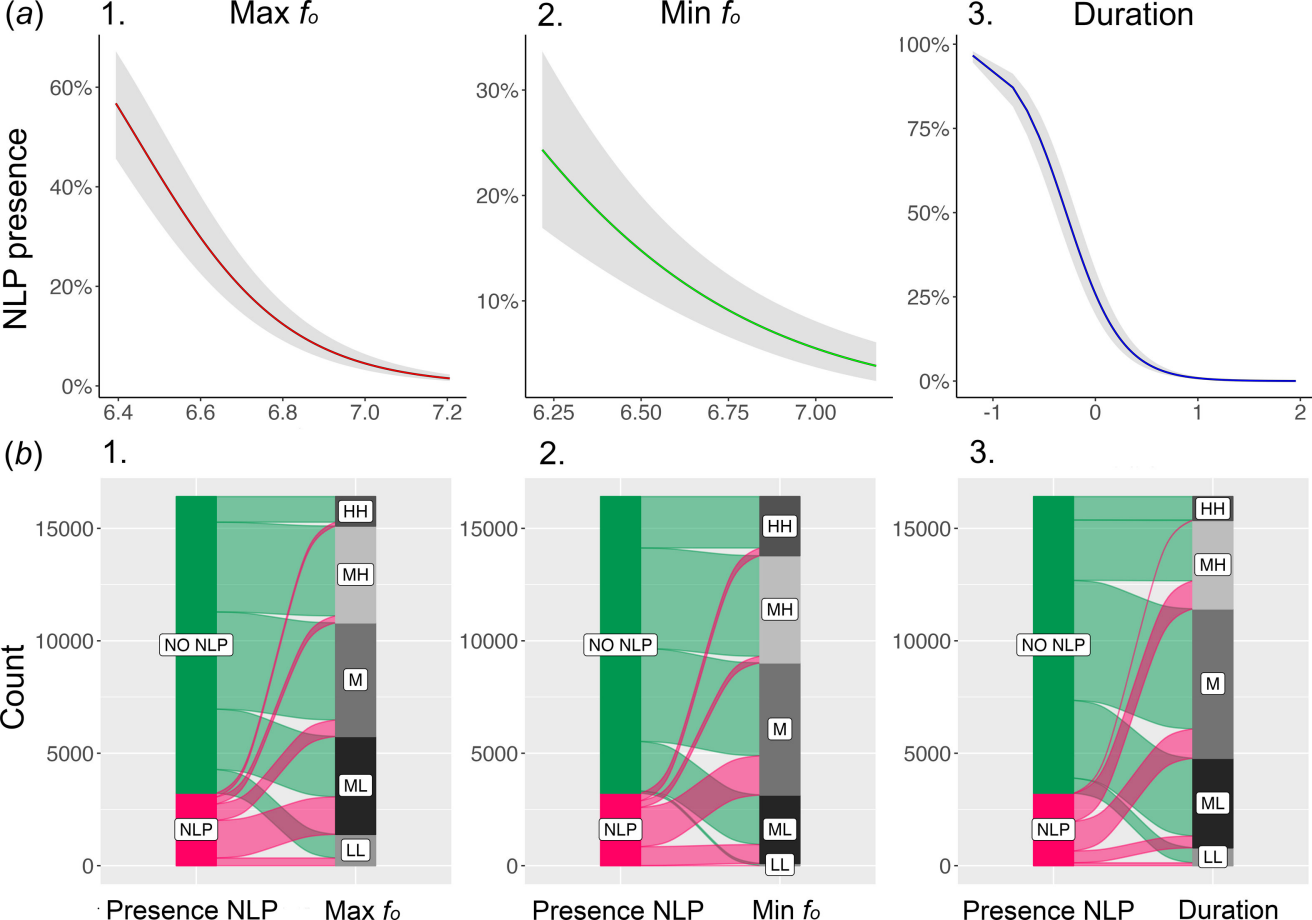
Relationship between presence of NLP and spectro-temporal notes’ parameters. parameters. (*a*) Plots of the model results showing the relationship between the response variable (presence of NLP) and the fixed factors (i) unit’s maximum *f*_o_, (ii) unit’s minimum *f*_o_, (iii) unit's duration (s). (*b*) Alluvial plot showing the presence (NLP) or absence (NO NLP) of NLP in a unit, and (i) unit’s maximum *f_o_*, (ii) unit’s minimum *f_o_*, (iii) unit's duration (s). In each panel, the vertical sizes of the first blocks are proportional to the number of notes containing or not containing NLP. The thickness of each stream shows the proportion of observations belonging to each stratum of the second block. The second blocks display continuous variables that are discretized in five equally ranged bins by default by the package *easyalluvial*: LL, low low; ML, medium low; M, medium; MH, medium high; HH, high high. We employed the R packages *ggplot2* [57], *ggeffects* [58] and *easyalluvial* [59] for visualizing data.

We found that units containing NLP had lower values of maximum (figure 3*a*.1, B.1) and minimum fundamental frequency (*p* < 0.001, for both cases; electronic supplementary material, table S4; figure 3*a*.2, B.2) than units without vocal nonlinearities and were also shorter (*p* < 0.001, electronic supplementary material, table S4; figure 3*a*.3, B.3) when compared with units without NLP. The full model differed from the null one (χ^2^ = 12551.77, df = 4, *p* < 0.001).

#### (ii) Occurrence of different types of nonlinear phenomena based on note duration, maximum and minimum fundamental frequency

#### *Maximum f*_*o*_

**Figure 4.**
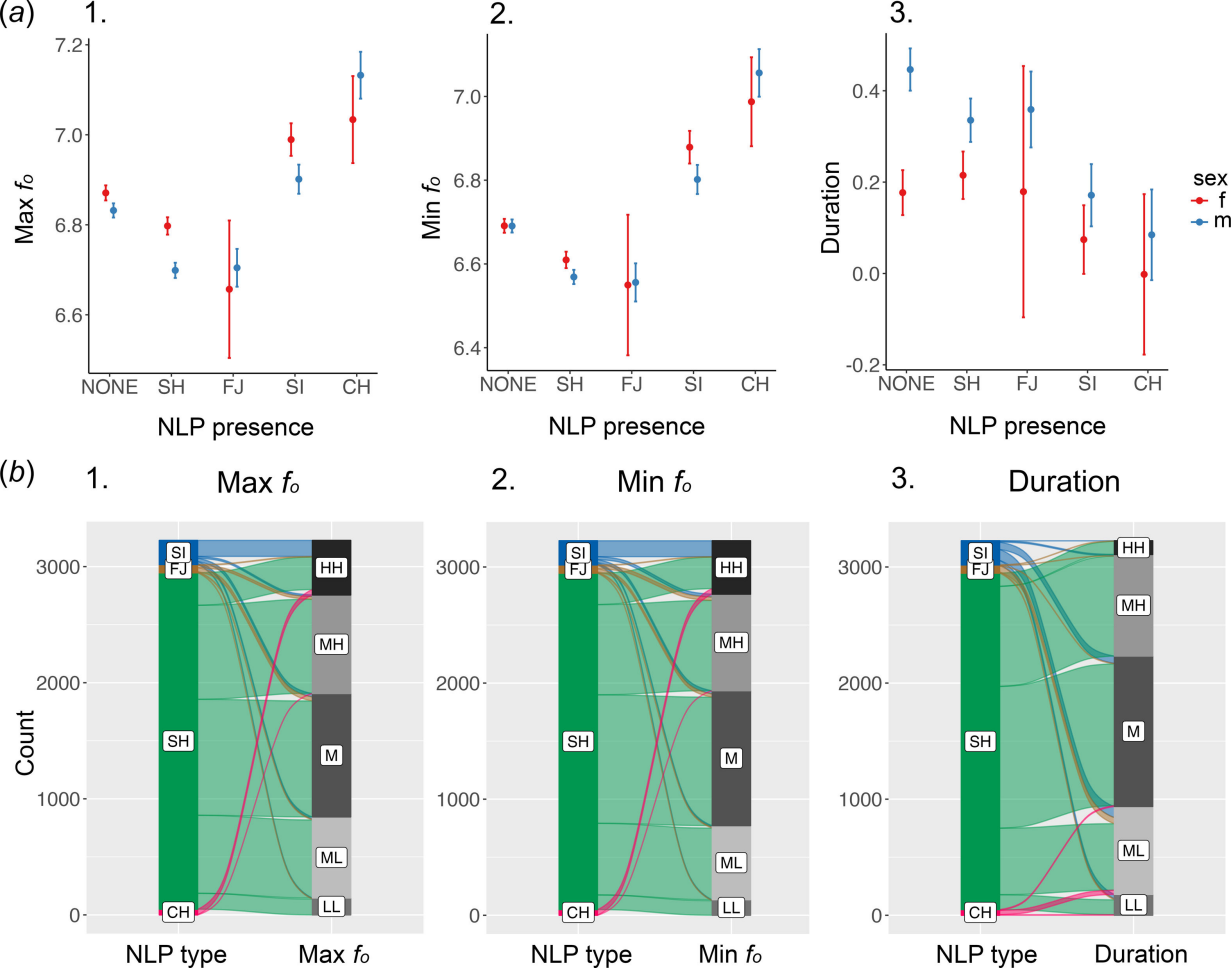
Relationship between the type of NLP and spectro-temporal notes’ parameters. (*a*) Plots of the model results showing the relationship between the response variable (type of nonlinear phenomenon) and (i) unit’s maximum *f_o_*, (ii) unit’s minimum *f_o_*, (iii) unit's duration. (*b*) Alluvial plot showing the type of nonlinear phenomena (NLP) in a unit and (i) unit’s maximum *f_o_*, (ii) unit’s minimum *f_o_*, (iii) unit's duration. In each panel, the vertical sizes of the first blocks are proportional to the number of NLP types. The thickness of each stream shows the proportion of observation belonging to each stratum of the second block. The second blocks display continuous variables that are discretized in five equally ranged bins by default by the package *easyalluvial*: LL, low low; ML, medium low; M, medium; MH, medium high; HH, high high. We employed the R packages *ggplot2* [57], *ggeffects* [58] and *easyalluvial* [59] for visualizing data.

The full model differed from the null one (χ^2^ = −14763.26, df = 9, *p* < 0.001). The *post hoc* results showed that units without NLP had higher values of maximum fundamental frequency than units containing subharmonics (*p* < 0.001) and frequency jumps (*p* < 0.001; figure 4A.1). On the other hand, units with sidebands and deterministic chaos had a higher maximum fundamental frequency than units not containing vocal nonlinearities (*p* < 0.001, in both cases; electronic supplementary material, table S5; figure 4B.1). Units with chaos had the highest maximum fundamental frequency, followed by units containing sidebands. Units with subharmonics had a lower value of maximum fundamental frequency than those showing sidebands. Units containing frequency jumps showed the lowest values (electronic supplementary material, table S5; figure 4A.1). Moreover, the *post hoc* comparison on the interaction between NLP type and sex showed that notes containing subharmonics and sidebands had higher maximum fundamental frequency in females than males (*p* < 0.001 and *p* = 0.014, respectively; electronic supplementary material, table S5; figure 4B.1).

#### *Minimum f*_*o*_

The full model differed from the null one (χ^2^ = −11606.62, df = 9, *p* < 0.001). The *post hoc* results showed that units without NLP had higher values of minimum fundamental frequency than units containing subharmonics (*p* < 0.001, electronic supplementary material, table S5; figure 4*a*.2,B.2) and frequency jumps (*p* = 0.015). Differently, units without NLP had lower minimum fundamental frequency than units displaying chaos (*p* < 0.001, electronic supplementary material, table S5). Units with chaos had the highest minimum fundamental frequency, followed by units containing sidebands (electronic supplementary material, table S5; figure 4*a*.2, B.2). Units with subharmonics had a lower value of minimum fundamental frequency than those showing sidebands. Units containing frequency jumps showed the lowest values (electronic supplementary material, table S5; figure 4*a*.2, B.2). Moreover, the *post hoc* comparison of the interaction between NLP type and sex showed that notes containing subharmonics tend to have higher values of minimum fundamental frequency in females than males (*p* = 0.066; electronic supplementary material, table S5).

#### Unit duration

The full model differed from the null one (χ^2^ = 4024.667, df = 9, *p* < 0.001). The *post hoc* results showed that units without NLP were longer than units containing them (electronic supplementary material, table S5; figure 4*a*.3,B.3). Units containing subharmonics were also longer than the ones containing chaos (*p* < 0.001) and sidebands (*p* < 0.001). Moreover, the *post hoc* comparison of the interaction between NLP type and sex showed that notes without NLP and those containing subharmonics had longer duration in females than males (*p* < 0.001 and *p* = 0.027, respectively; electronic supplementary material, table S5; figure 4*a*.3,B.3).

### (d) Short-term vocal fatigue: notes position and presence and duration of nonlinear phenomena

#### (i) Duration of nonlinear phenomena

The full model differed from the null one (χ^2^ = 115.525, df = 7, *p* < 0.001). We found that NLP occurring at the end of a phrase last position = "L") lasted longer than (i) those occurring at the beginning (first position = "F") of the phrase (F vs L, *p* < 0.001; electronic supplementary material, table S6), (ii) in the middle (intermediate position = "I") phrase (I vs L, *p* < 0.001, electronic supplementary material, table S6), and (iii) in single units and long notes (L vs M, *p* = 0.003; electronic supplementary material, table S6). On the other hand, males and females did not differ in terms of NLP duration based on note position (electronic supplementary material, table S7).

#### (ii) Occurrence of nonlinear phenomena based on note position

The full model differed from the null one (χ^2^ = 12186.84, df = 7, *p* < 0.001). The *post hoc* comparison showed that notes occurring in the last position of a phrase had the highest probability of containing NLP (electronic supplementary material, table S8), followed by notes in the intermediate position, and then by notes that are not part of a phrase, and, finally, by notes occurring in the first position of a phrase, which had the lower probability of containing NLP.

Finally, the *post hoc* comparison on the interaction between note position and the sex of the emitter showed that females have more NLP occurring in the last note of a phrase than males (*p* < 0.001, electronic supplementary material, table S9). At the same time, we found no differences for other note positions.

## 4. Discussion

In our study, we investigated the occurrence of NLP in the songs of the indri, the only lemur species exhibiting singing behaviour. We found that the indris’ songs contained four different types of NLP (subharmonics, frequency jumps, deterministic chaos and sidebands) and that the occurrence of these phenomena changed during ontogeny. We also found that notes containing vocal nonlinearities were shorter and had a lower fundamental frequency than those without NLP. Finally, our results support the presence of short-term vocal fatigue, as not only do notes occurring in the last position of a phrase have the highest probability of containing NLP, but NLP in these positions also lasts longer.

### (a) Effect of sex on the presence and type of nonlinear phenomena

We found that vocal nonlinearities in the indris’ songs increased with age and that males’ notes contained more NLP than females’. This result is partially in line with what was previously found by Cristiano and colleagues [33], namely, that adults emit more NLP than juveniles but with no differences between males and females. This discrepancy could be related to the difference in song and individual sample size, which, in our case, was more than three times (for songs) and more than twice (for individuals) the number used in the previous work on this topic [33].

Why do male indris have more vocal nonlinearities than females? There are two possible, non-mutually exclusive hypotheses. The first one concerns their physiology. Previous research found that females show a higher degree of vocal flexibility than males [39,44,60,61] and, therefore, we can hypothesize males to have poorer control of the phonatory apparatus. Studies on humans found that the female vocal tract is better for speech than the male one, having the ability to produce a broader range of acoustic signals [62]. One of the potential explanations for this sexual dimorphism resides in the selective pressure that led to the lowering of the male larynx, namely the theory of size exaggeration [62–64]. It is unclear whether this particular explanation aligns with the indris’ vocal behaviour. Still, some form of selective pressure likely also acted on the structure of males’ vocal units. Therefore, our second hypothesis involves sexual selection, the primary evolutionary cause of sex difference [65]. Indris live in a female-dominated society where female mate choice and intra-sexual male selection are likely to take place [66]. Males are the primary actors in the physical defence of the family group [67]. It might be advantageous for males to exhibit more vocal nonlinearities within a vocalization primarily aimed at neighbouring social groups and floating individuals [38,43]. NLP are known to attract attention and convey urgency [13,14] and, in humans, also enhance the perception of both positive and negative emotional states [20]. Female red deer (*Cervus elaphus*) paid substantially more attention to male sexual calls containing NLP than those without vocal nonlinearities, and they maintained the attention during the subsequent calls with no NLP [68]. Therefore, a higher occurrence of vocal nonlinearities might have been positively selected in males for their role in boosting the communicative valence of singing behaviour, conveying urgency or attracting listeners’ attention.

### (b) Effect of age on the presence and type of nonlinear phenomena

In particular, we observed that the indris produced four types of NLP: subharmonics, which were the most common, followed by frequency jumps, deterministic chaos and sidebands. We found further sex-related differences in the proportion of NLP types and their ontogenetic variation. When considering the quantity of NLP, we saw increased vocal nonlinearities with growth. Still, considering their type, we found different developmental trajectories between subharmonics and the other NLP. The high taxon heterogeneity of studies addressing the same topic means that caution is required in framing our results in the current literature. Indeed, NLP may vary in both production mechanisms and function across phylogenetically distant species. Yet, some parallels with previous research can still be drawn. On the one hand, a higher occurrence of NLP with growth might be a potential ageing indicator, as suggested for domestic dogs [26] and African penguins (*Spheniscus demersus*) [28]. On the other hand, the fact that deterministic chaos, sidebands and frequency jumps are more common in younger individuals might (i) provide a vocal cue on the age of the emitter and (ii) suggest that, in individuals having a not fully developed vocal apparatus, deterministic chaos, sidebands and frequency jumps might occur more than subharmonics, which, in turn, remain stable during growth. Similarly, Volodin *et al*. [24] found that different types of NLP occurred at various developmental stages in steppe lemmings (*Eolagurus luteus*), suggesting a potential role of vocal nonlinearities in providing information on the animal’s age.

### (c) Different types of nonlinear phenomena are associated with different vocal constraints

We found that notes containing vocal nonlinearities were shorter and had a lower maximum and minimum fundamental frequency than notes without NLP. Still, we found different trends when considering the various types of NLP produced. We also found that notes without NLP were longer than notes containing all types of nonlinearities, except for frequency jumps, which were present regardless of the notes’ duration. This result is in line with previous work that highlighted, in the indris’ songs, the adherence to laws of compression, namely that the duration of a unit decreases with the increase of the phrase size [69]. Therefore, NLP may occur in shorter units because they belong to longer phrases, where vocal fatigue is more likely to occur [33]. Notes containing subharmonics and frequency jumps have lower maximum and minimum fundamental frequencies than notes without NLP, while notes containing chaos and sidebands had higher values. Keeping in mind that the faster the vocal folds’ vibrations, the higher the pitch of the sound they generate, our result suggests that different types of NLP are associated with different vocal constraints: chaos and sidebands may arise when vocal folds vibrate faster than when producing units with no NLP. At the same time, subharmonics occur when vocal folds' vibration is too low. This is also in line with young individuals producing a higher proportion of chaos and sidebands: an underdeveloped vocal apparatus might have poorer vocal control on the fast tempo of vocal fold vibration.

We found that females had a higher proportion of sidebands compared with males. Different factors could contribute to this difference. First, females emit more notes and phonate longer than males during singing events [60]. The occurrence of more sidebands in females might be the by-product of temporary vocal fatigue and the greater difficulty associated with producing higher-pitched notes than regular ones. At the same time, like sidebands, chaos also appeared in higher-pitched notes, but there were no differences between the sexes. Therefore, a second explanation could be related to the mechanism of sideband production: sidebands can be defined as a type of biphonation occurring when one source vibrates at a much lower frequency than the other [34], and biphonation has been considered a mechanism to increase call complexity, which can be used, for example, in individual or group recognition [7,70,71] or to advertise body size [72]. We speculate that similar mechanisms are present in the indris' songs, where female indris are more prone than males to produce units with a more complex structure, as they possess greater vocal flexibility and plasticity in vocal output than males [39,60,61].

### (d) The case of subharmonics

It is intriguing that subharmonics are the more common type of NLP in the indris’ songs and that they are the only type of NLP that remains stable through ontogeny. A first explanation might be that low-pitched notes containing subharmonics are challenging to produce at all ages. However, the proportion of frequency jumps, also present in low-pitched notes, decreases with age. Therefore, we cannot dismiss a second possibility: subharmonics serve a purpose in indris' songs, which is in line with other studies suggesting a functional role of this type of NLP in animal communication. For example, subharmonics in the calls of female concave-eared torrent frogs (*Odorrana tormota*) elicit different vocalizations from their male counterparts compared with normal calls [73]. Similarly, female koala (*Phascolarctos cinereus*) rejection calls containing more subharmonics attract male interest [72]. We can hypothesize that subharmonics might play a functional role by making signallers appear larger by lowering the perceived frequency, as suggested by Fitch and colleagues [3], and also because subharmonics appear in notes with the lowest minimum value of fundamental frequency. Indris live in a densely forested habitat where visual contact with neighbouring groups is rare [42], and from songs’ features indris derive important information such as sex, age and neighbour identity [43,60,74]. Notes containing subharmonics are the ones with the lowest frequency and, therefore, whose sound travels further [75]. Moreover, juvenile and subadult individuals sing in the chorus alongside their parents during territorial confrontations [76] and advertise their presence to neighbours during morning advertisement songs [31]: in young animals, as in adults, low-frequency notes and subharmonics might be used as cues to inform neighbouring groups of their presence and physical features. Thus, the presence of subharmonics in the indris’ songs might be balanced between the possibility of appearing more intimidating to potential rivals and the potential costs associated with the production of vocal nonlinearities [8].

### (e) Short-term vocal fatigue

We found that the position of a unit within a phrase influences the presence and duration of NLP. Specifically, we observed that NLP last longer when emitted at the end or in the middle of a phrase than when emitted at the beginning. Furthermore, in line with Cristiano *et al*. [33], we found that at the phrase level, nonlinearities are more likely to occur in the final stages of the vocal display [33]. Such findings, therefore, strengthen the hypothesis that indris may experience short-term vocal fatigue while singing. Like in other primates [34], including humans [35], intense vocal activity may involve some degree of vocal fatigue eliciting NLP. In this context, physiological constraints may partially impair vocal emission, resulting in vocal fatigue, which could be considered the cost of performing loud, long and complex vocal performances. We also provided evidence that there is a higher probability that the last note of a phrase is nonlinear in females than in males, given that NLP occur more often in the last unit of a female phrase. This difference between the sexes might reflect the sexual dimorphism occurring in the distinct engagement of females and males during the song. Indeed, females show longer individual contributions and shorter intervals between the onsets of two consecutive units [60] and emit more units and phrases than males [61,74].

Moreover, previous findings showed that adult females tend to emit the first nonlinear phenomenon earlier during the song than do adult males [33]. These findings suggest that although male notes contain more NLP overall, females may be more prone to short-term vocal fatigue during the song.

## 5. Conclusions

Our research supports the role of physiological constraints in shaping NLP presence, as (i) notes containing vocal nonlinearities reach pitches that are higher or lower than the ones that characterize notes without NLP, (ii) the developmental stage of the vocal apparatus influences the presence of NLP, which occur more in younger animals, and (iii) the presence of short-term vocal fatigue might explain why notes containing nonlinearities occur later in a phrase composed of multiple notes. On the other hand, we also found indications that NLP might also have a functional role in indris’ communication: sexual dimorphism in the occurrence of NLP, together with the fact that subharmonics do not change differentially by age, suggest that other selective pressure might have shaped the presence of vocal nonlinearities in the song of non-human primates. This interpretation aligns with different studies reporting that the perturbation of the harmonic structure of sounds represents a source of acoustic variation that can encode information about the emitter. The implications of our findings transcend the boundaries of species-specific explanations for the presence of NLP in vocal production, offering insights into the evolutionary roots of vocal nonlinearities in the animal kingdom.

## Data Availability

The datasets supporting this article have been uploaded as part of the electronic supplementary material. Supplementary material is available at [77].
